# Tolerance of chickens to acute and sub-acute intravenous administration of raw filtered honey

**DOI:** 10.3389/fvets.2026.1832182

**Published:** 2026-07-14

**Authors:** Madison Morgan, Joerg Mayer, Chloe Goodwin, Karen Grogan, Frane Banovic, Robert M. Gogal

**Affiliations:** 1 Department of Small Animal Medicine and Surgery, University of Georgia, College of Veterinary Medicine, Athens, GA, United States; 2 Department of Biomedical Sciences, University of Georgia, College of Veterinary Medicine, Athens, GA, United States; 3 Department of Pathology, University of Georgia, College of Veterinary Medicine, Athens, GA, United States; 4 Department of Population Health, Poultry Diagnostic and Research Center, University of Georgia, College of Veterinary Medicine, Athens, GA, United States

**Keywords:** apitherapy, chicken, histology, immune response, intravenous administration

## Abstract

**Objective:**

This study evaluated tolerance and immune response in 30 chickens following intravenous (*IV*) administration of filtered, raw honey.

**Methods:**

In the acute trial, 10 birds (one male and one female per dose) received a single *IV* infusion of honey at 0.0, 1.0, 2.0, 3.0, or 4.0 mL/kg and were monitored for 7 days. In the sub-acute trial, 20 birds received three *IV* infusions at 0.0, 1.0, or 3.0 mL/kg at 48-h intervals over 5 days, with at least six birds per treatment group, and were monitored for 22 days. At the end of each trial, birds were bled, euthanized, necropsied, and tissues (gonads, heart, liver, lung, kidney, spleen and vein) were collected for histopathology.

**Results:**

No adverse clinical signs, significant gross lesions, or histopathological changes attributable to treatment were observed. No differences were found in plasma chemistry, peripheral blood cytology, leukocyte immunophenotypes, or cytokine levels between honey-treated birds and controls in the sub-acute trial.

**Conclusion:**

Chickens tolerated acute *IV* doses up to 4 mL/kg and multiple sub-acute infusions up to 3 mL/kg of raw, filtered honey without overt adverse systemic effects under the study conditions.

**Clinical relevance:**

Apitherapy using honeybee products is an emerging alternative treatment. However, safety and efficacy data, particularly for intravenous administration in avian species, remain limited.

## 1 Introduction

The therapeutic use of honeybee products, collectively termed apitherapy, has gained acceptance in both traditional and modern medicine (1, 2). Honeybee products include honey, bee venom, propolis, royal jelly, pollen, and beeswax. Apitherapy, including nutraceutical applications, has experienced renewed interest as part of integrative and complementary medicine (3, 4). Recent studies have demonstrated immunomodulatory properties of these products in several veterinary species (5). Honey promotes wound healing and serves as an adjunct therapeutic in select systemic diseases because of its antibacterial and anti-inflammatory effects (6, 7). Its antibacterial activity arises from low pH, high osmolarity, hydrogen peroxide content, and bee defensin-1 (8). Additional research indicates anti-inflammatory and antineoplastic properties, along with promotion of epithelialization and tissue healing (3, 9). In humans, tolerance to *IV* honey was evaluated in 140 healthy and unhealthy volunteers (10). Patients of varying ages and health statuses tolerated *IV* honey without severe systemic reactions (10).

In veterinary medicine, *IV* honey has been studied in mammals. Slow *IV* honey administration (15 drops/min) in sheep was well tolerated and improved renal, hepatic, and bone marrow function (11). In female goats, multiple daily *IV* honey infusions (70–80 drops/min) increased antioxidant parameters and decreased free radical metabolites (12). In a mouse melanoma model, *IV* honey reduced tumor growth and improved survival when combined with chemotherapy (13). Honey may inhibit carcinogenesis by modulating the molecular processes in cancer cell initiation, promotion, and progression stage (14). Thus, with additional studies, apitherapy might be viewed as a valuable adjunct therapy for cancer patients who are undergoing chemotherapy, based on its nonspecific antineoplastic properties.

Apitherapy in avian species remains largely unexplored. Pet birds frequently present with aggressive neoplasia, including lymphoma, adenocarcinoma, and hematopoietic neoplasms, of which approximately 79% are malignant (15). Psittaciformes, a common exotic pet, are commonly affected (16).

From a therapeutic point of view, surgical options are often limited by anatomy, tumor location, and undetected metastasis (17). Birds exhibit high metabolic rates that may accelerate tumor progression and relative resistance to radiation, necessitating higher doses and more frequent treatments (16–18). Chemotherapy carries risk of gastrointestinal toxicity and myelosuppression (19). *IV* honey could potentially serve as a low-toxicity adjunct in avian oncology.

This study assessed whether chickens tolerate filtered raw honey administered *IV* at varying doses acutely and sub-acutely, and whether immunity is affected. The acute phase evaluated single infusions up to 4 mL/kg over 7 days. The sub-acute phase evaluated three infusions (0, 1, or 3 mL/kg) over 5 days with monitoring for 22 days. To our knowledge, this is the first *in vivo* study of *IV* filtered-honey administration in an avian species.

## 2 Materials and methods

### 2.1 Honey collection and solution preparation

Honey was harvested in the summer of 2024 from hives at the UGA Veterinary Teaching Hospital. Frames were centrifuged in a commercial 3-frame tangential honey extractor, then filtered through a commercial 300 μm filter (Gefuka®), and transferred to a glass bottle. One mL of filtered honey was mixed with 2 mL of sterile saline to create the honey solution. This solution was passed through an 18 μm filter (Utah Medical) into a sterile *IV* set (Zoetis) and administered within 10 min of preparation.

### 2.2 Chickens

Thirty 6-week-old broiler chickens (male and female; 2.35 kg to 3.63 kg) were obtained as extra negative-control birds from a completed poultry nutrition study at the University of Georgia Department of Poultry Science. Birds were housed in isolator units (2 birds/cage) and arbitrarily assigned to acute (*n* = 10) or sub-acute (*n* = 20). Lighting was 12 h on/12 h off. Ammonia gas was not measured within the isolator cages. Feed and water were provided *ad libitum*. All procedures were approved by the University of Georgia IACUC (AUP A2023 08-30-Y1-AO).

### 2.3 Acute phase trial

**Figure 1:**
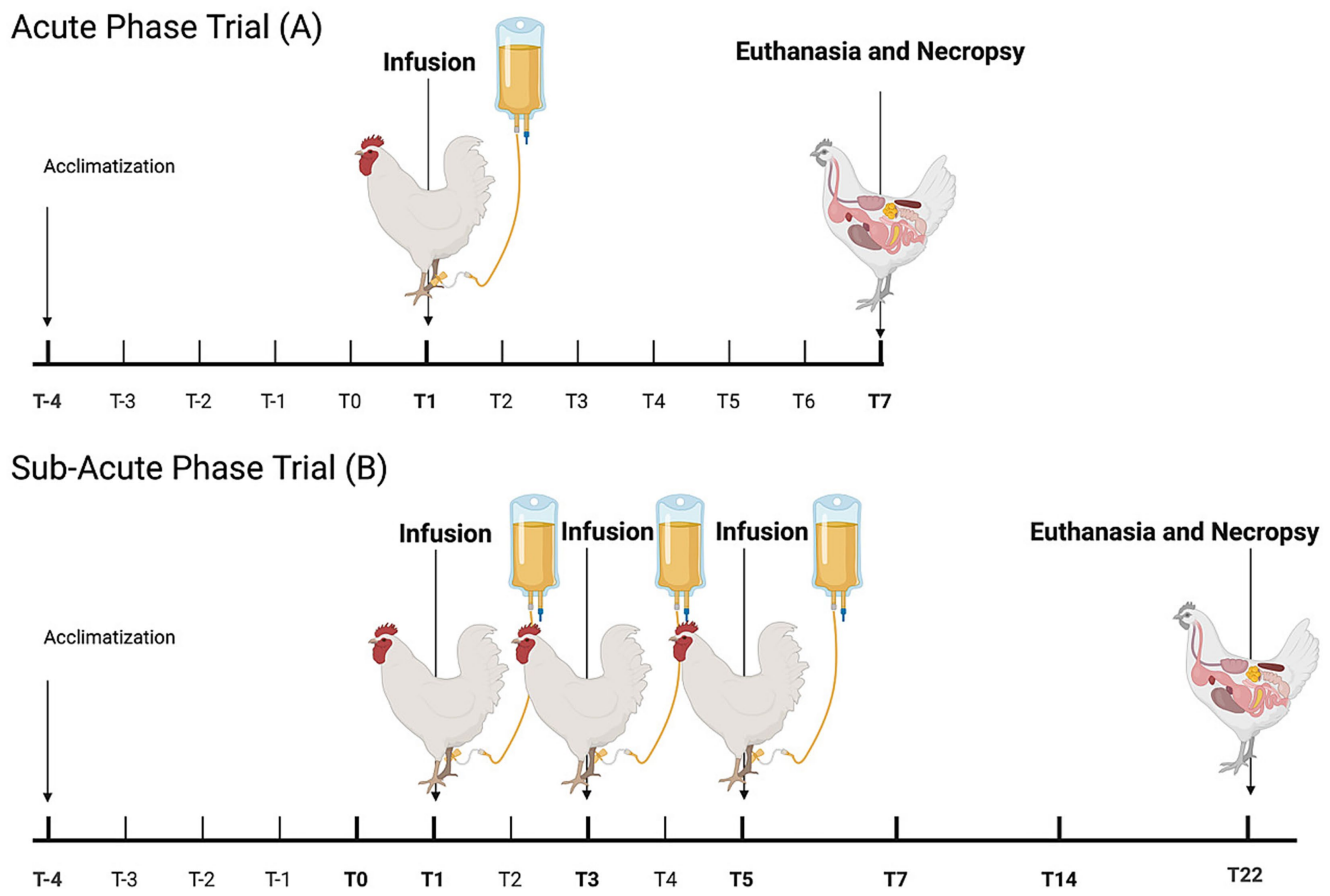
Injection event timeline. Birds (*n* = 5 birds/sex) in the acute phase trial were acclimated for 4 days then received a single *IV* infusion of saline or filtered honey (1, 2, 3, or 4 mL/kg per bird/sex) on T1 and then euthanized on T7 **(A)**. Birds (*n* ≥ 6 birds/treatment) in the sub-acute phase were acclimated for 4 days then received multiple *IV* infusions of saline or filtered honey (1, 2, or 3 mL/kg per bird) on T1, T3, and T5, and euthanized on T22 **(B)**. This image was generated using the BioRender software.

Ten chickens (5 males, 5 females) were assigned to five treatment groups (1 male + 1 female/group): control (saline), 1 mL/kg, 2 mL/kg, 3 mL/kg, or 4 mL/kg of honey solution. Infusions were delivered via the medial metatarsal vein using a 23G butterfly catheter and syringe pump. Doses of 1 and 2 mL/kg were infused over 10 min; doses of 3 and 4 mL/kg were infused over 20 min to reduce fluid-overload risk. All birds were sedated 10 min prior with midazolam (3–4 mg/kg IM) and butorphanol (3–4 mg/kg IM) into the pectoral muscles, with higher doses used for longer infusions. Birds recovered without reversal. Daily clinical observations were performed. On day 7, birds were euthanized by cervical dislocation. Tissues (liver, kidneys, lungs, spleen, heart, gonads, medial metatarsal vein) were collected for histopathology (Figure 1).

### 2.4 Sub-acute phase trial

A total of 20 chickens (10 male and 10 female) were used in the sub-acute phase trial. The control-saline group consisted of 6 chickens: 3 male and 3 female. The 1 mL/kg dose group consisted of 7 chickens: 4 male and 3 female. Lastly, the 3 mL/kg dose group consisted of 6 chickens: 3 male, 3 female. Each group received 3 infusions every 48-h over a 5-day period. The first infusion was over 10 min for all dose groups. The second and third infusions were administered over 10 min for the control and 1 mL/kg dose groups, and over 20 min for the 3 mL/kg dose group, due to the large volumes being injected. Similar to the acute study, all birds were sedated 10 min prior to infusions via an IM injection into the pectoral muscles with midazolam at 2 to 4 mg/kg and butorphanol at 2 to 4 mg/kg, depending on their sedation depth after 10 min. No reversals were administered.

Birds were monitored daily for adverse clinical reactions for 18 days post final infusion. Weight data were collected at day 1 prior to infusions, day 5 prior to infusions, and day 22 prior to euthanasia. Peripheral blood (3–5 mL) was collected prior to the first infusion and prior to euthanasia. The following assays were performed: plasma chemistries, whole blood 5-point differentials, cytospins, peripheral blood leukocytes cell marker phenotypes, and peripheral blood plasma cytokine levels. All birds were euthanatized with Pentobarbital (2 mL/kg *IV*) administered via the metatarsal vein, 18 days after the last honey infusion. Necropsies were performed post euthanasia, and the following tissues were collected for histopathology: liver, kidneys, lungs, spleen, heart, gonads, medial metatarsal veins, and feet scabs (Figure 1).

### 2.5 Blood collection

Peripheral blood was collected during the sub-acute phase trial from the medial metatarsal vein on day 1 prior to infusion and on day 22 prior to euthanasia. Approximately 3–5 mL of blood were collected with a 23GA butterfly catheter and 6 mL syringe. Blood was transferred into a 4 mL heparinized green top tube and a lithium heparin microtainer, then gently inverted and stored at 4 °C until processing.

### 2.6 Peripheral blood leukocyte enrichment

The peripheral blood heparinized green top tubes were removed within 12–16 h of collection from 4 °C storage and allowed to slowly reach 23 °C (20, 21). Each tube was centrifuged twice at 40xg, 10 min, 23 °C (0 accel, 1 decel). The buffy coat, located between the plasma and RBC interface, was carefully collected from each tube using a “swirl technique” (22) and transferred to a sterile 15 mL conical tube (Thermo Fisher Scientific). Each tube was centrifuged at 400xg, 10, 7 °C (7 accel, 7 decel). The plasma was collected into microfuge tubes and frozen at −80 °C until analyzed for cytokine levels. The cell pellets were resuspended in 5 mL incomplete RPMI media (Corning, calcium and magnesium free) then centrifuged at 400xg, 10 min, 7 °C (7 accel, 7 decel). The supernatant was discarded, and each sample was resuspended in 3 mL of incomplete RPMI media (Corning). A 100 μL aliquot of each sample was collected and enumerated using the Cellometer™ Auto T4 (Nexcelom). The peripheral blood leukocytes (PBLs) were resuspended 4.0 × 10^6^ cell/mL in complete media. Leukocyte viability at collection was >80%.

### 2.7 Plasma chemistries

During each blood draw, 500 μL of blood were placed into a lithium heparin microtainer, then stored at 4 °C until analysis on the same day. Chemistries were evaluated on the day of blood collection using a Vetscan chemistry analyzer with avian/reptilian profile plus rotors. Rotors were stored in the refrigerator until use. Once rotors were removed from the refrigerator, 100 μL of peripheral blood was pipetted into the sample port and the rotor was placed into the analyzer.

### 2.8 Peripheral blood leukocyte cytology

Blood smears were made from the lithium heparin microtainers within 12 h of the blood draws. Smears were stained with a Wright-Giemsa stain using a 10-min full stain/10-min diluted stain protocol. After the slides were stained and dried, they were mounted and viewed under 100x oil. A total of 100 leukocytes (heterophils, lymphocytes, monocytes, eosinophils and basophils) were enumerated over a minimum of 10 microscopic fields. Values were expressed as a percentage out of 100 leukocytes.

### 2.9 Peripheral blood leukocyte cytospin

Cytospins were made from enriched peripheral blood cell suspensions (5.0 × 10^5^/mL). Each sample was suspended to a volume equivalent to 6 × 10^4^/chamber cup. The cytospin chambers were attached to vertically mounted slides and placed in an 8 chamber cytospin rotor. Slides were allowed to dry overnight, then stained using a modified Wright-Giesma stain protocol of 10-min full stain/10-min diluted stain. Each slide was rinsed with water and allowed to air dry prior to coverslip mounting. Slides were evaluated under 100x oil.

### 2.10 Peripheral blood leukocyte cell surface marker phenotypes

In order to evaluate the differences in select leukocyte sub-populations in the peripheral blood, 5.0 × 10^5^ cells/well were stained with anti-chicken CD8, Mono and IgM (0.5 mg/5.0 × 10^5^ cells, Southern Biotech) antibodies for 30 min at 4 °C in the dark. Afterwards, the cells were washed with 1X concentrated phosphate-buffered saline (PBS; HyClone) and centrifuged at 200xg for 10 min at 4 °C. The cell pellets were then resuspended with 100 mL of 1X PBS and fixed with 100 mL of 2% paraformaldehyde. Samples were evaluated on a BD-LSR II flow cytometer measuring 10,000 events per sample. Values were reported as percent expression.

### 2.11 Peripheral blood plasma 12-plex cytokine panel

Peripheral blood plasma samples from Day 1 (Baseline) and Day 22 (post *IV* honey) were evaluated for changes in 12 chicken cytokine (IL-16, IFNa, IFNg, IL-10, IL-21, IL-2, M-CSF, IL-6, MIP-3a, MIP-1b, Rantes, and VEGF) levels using a chicken MILLIPLEX Px12 kit (EMD Millipore Corporation). Briefly, 25 mL of standards, diluted sera and supernatants were aliquoted into select wells of a 96-well plate, which was followed by the addition of 25 mL of pre-mix beads or matrix buffer and then incubated overnight (18 h) on a shaker at 4 °C. Plates were washed and received 25 mL detection antibody and incubated 60 min at 23 °C. To each well, 25 mL of Streptavidin-PE was added and incubated for 30 min at 23 °C. The plates were then washed, and the wells resuspended in 150 mL sheath fluid and enumerated on a Luminex 200 (Luminex Corporation) and analyzed with XPONENT software solutions (Luminex). The values were reported in pg/mL.

### 2.12 Histopathology

A standardized necropsy procedure followed euthanasia. The gonads, heart, liver, lung, kidney, spleen, metatarsal vein, and any tissues with gross lesions were collected and fixed in 10% neutral-buffered formalin. Tissues were trimmed and routinely processed prior to embedding in paraffin, sectioning at 5 μm thick, and affixed to glass slides. All slides were stained with hematoxylin and eosin prior to histologic examination. The slides were evaluated by a board-certified pathologist who was blinded to the treatment groups for both the acute and sub-acute phases trials. Digital images were generated, and tissues were lesion scored based on 5 levels from no lesions to severe.

### 2.13 Statistics

Statistical analyses used GraphPad Prism v10.5.0 (GraphPad Software) with two-way mixed effects models (*p* ≤ 0.05). Body weights, cytokine and chemokines, leukocyte cell surfaces markers, cytology, plasma chemistries, and cytospin values within and between treatment groups were analyzed. Bonferroni’s or Tukey’s correction tests were used for multiple comparisons between all groups of values. Data are expressed as median, minimum value, and maximum value. Normality testing using Shapiro–Wilk test was calculated for all samples and groups. Findings considered statistically significant with *p*-values ≤ 0.05 are indicated with alphabetical superscripts.

## 3 Results

### 3.1 Histopathology: acute trial

Mild pericardial effusion occurred 5/10 chickens (50%), including 2 saline controls. Heart lesions included focal to multifocal fibrosis (4/10; 40%) and subintimal fibrosis of medium-sized arterioles (2/10; 20%). No significant treatment-related histopathologic lesions were observed in the liver, lungs, or kidneys (Table 1; Supplementary Figure S1).

**Table 1:** Histopathology scores.

IV Honey trial	Dosage	Chicken	Heart	Liver	Lung	Kidney	Spleen	MT vein
Acute phase trial	Control	Female	−	−	−	−	−	−
		Male	++	−	−	n/a	−	−
	1 mL/kg	Female	−	−	−	n/a	−	−
		Male	−	−	−	−	−	−
	2 mL/kg	Female	−	−	−	−	−	−
		Male	++	−	+	−	−	+
	3 mL/kg	Female	++	−	−	−	−	−
		Male	−	−	−	−	−	+
	4 mL/kg	Female	+	−	−	−	−	−
		Male	−	−	−	−	−	−
Sub-acute phase trial	Control	Female	−	−	−	−	−	−
		Female	++	−	−	−	−	−
		Female	−	−	−	−	−	+
		Male	++	−	−	−	−	−
		Male	−	−	−	−	−	−
		Male	−	−	−	−	−	−
	1 mL/kg	Female	−	−	−	−	−	−
		Female	−	−	−	−	−	−
		Female	−	−	−	−	−	−
		Male	−	−	−	−	−	−
		Male	++	−	−	−	−	−
		Male	−	−	−	−	−	−
		Male	−	−	−	−	−	−
	3 mL/kg	Female	−	−	−	−	−	−
		Female	−	−	−	−	−	++++
		Female	−	−	−	−	−	−
		Male	−	−	−	−	−	−
		Male	−	−	−	−	−	−
		Male	−	−	−	−	−	−

Lesion scoring	
−	No lesions
+	Minimal
++	Mild
+++	Moderate
++++	Severe

Histopathology lesion scores from the acute and sub-acute phase trials.

### 3.2 Body weight gain: sub-acute trial

All groups showed positive weight gain (Supplementary Figure S2). Between days 1–5, weight increased 15.60% (saline), 13.4% (1 mL/kg), and 9.4% (3 mL/kg). Between days 5–22, increases were 41.7% (saline), 47.0% (1 mL/kg), and 44.8% (3 mL/kg). Significant increases (*p* < 0.05 or *p* < 0.0001) occurred within groups as reported.

### 3.3 Plasma chemistries: sub-acute trial

Significant post-infusion increases in select analytes (UA, CA, TP, GLOB) were observed in all groups but were consistent with growth and diet (Table 2).

**Table 2:** Plasma chemistries: sub-acute trial.

Plasma chemistry values	Control		1 mL/kg		3 mL/kg	
	Pre-infusion Median (min–max)	Post-infusion Median (min–max)	Pre-infusion Median (min–max)	Post-infusion Median (min–max)	Pre-infusion Median (min–max)	Post-infusion Median (min–max)
AST (U/L)	359 (267–668)	608.5 (451–1,576)	332 (271–504)	756 (330–1,200)	300.5 (210–521)	528.5 (268–2,225)
BA (μmol/L)	0 (0–1)	2.5 (0–15)	0 (0–3)	3 (0–17)	0 (0–1)	4.5 (0–64)
CK (U/L)	0 (0–1,807)	139.5 (0–1,064)	0 (0–1,252)	66 (0–4,636)	772.5 (0–2,778)	0 (0–7,573)
UA (mg/dL)	3.5 (2.6–4.9)	**6**^**a**^ **(2.4–9.9)**	3.8 (2.5–4.8)	3 (1.5–5.8)	2.6 (1.8–3.1)	3.2 (1.8–6.3)
GLU (mg/dL)	252.5 (237–283)	226.5 (197–244)	222 (205–247)	217 (187–235)	225 (157–248)	235.5 (192–254)
CA (mg/dL)	10.3 (10–10.7)	**11.1**^**b**^ **(11–11.9)**	10.6 (10.2–11)	10.7 (10.5–11)	10.5 (6.9–10.9)	**10.9**^**c**^ **(10.1–11.6)**
PHOS (mg/dL)	6.9 (6.5–7.7)	6.8 (6.2–7.2)	6.5 (5.5–7.4)	6.3 (5.6–6.7)	6.9 (5.4–7.3)	6.7 (5.6–7.4)
TP (g/dL)	2.8 (2.7–2.9)	**3.7**^**d**^ **(3.4–4.6)**	2.8 (2.5–3.1)	**3.4**^**e**^ **(3.2–4.2)**	2.9 (2.1–3.3)	**3.7**^**f**^ **(2.7–4.4)**
ALB (g/dL)	2.2 (2.1–2.3)	2.6 (2.3–3)	2.1 (1.8–2.2)	2.2 (1.6–2.9)	2 (1.3–2.4)	2.3 (2–2.6)
GLOB (g/dL)	0.7 (0.5–0.9)	**1.3**^**g**^ **(0.8–2.1)**	0.7 (0.3–0.9)	**1.2**^**h**^ **(0.8–2.6)**	0.8 (0.7–1.1)	1.5 (0.7–1.8)
K + (mmol/L)	7.9 (2.8–8.6)	8.4 (7.6–8.8)	7.1 (4.9–9.2)	8.3 (5.3–8.8)	7.4 (6–8.3)	7.5 (2.4–8.6)
Na + (mmol/L)	149 (146–153)	148.5 (144–162)	148 (144–154)	146 (140–150)	150 (145–157)	149 (146–153)

Peripheral blood plasma chemistry values from the sub-acute phase trial. AST = aspartate aminotransferase, BA = bile acids, CK = creatine kinase, UA = uric acid, GLU = glucose, PHOS = phosphorous, CA = calcium, TP = total protein, ALB = albumin, GLOB = globulin, K+ = potassium, Na+ = sodium. ^a–h^Values with different superscripts are significantly different, *p* ≤ 0.05, Bonferroni’s test, Two-way mixed effects model, *N* = ≥6 birds/treatment. Values highlighted in bold were significant from control values.

### 3.4 Peripheral blood 5 point differential cytology: sub-acute trial

Blood smears were generated from peripheral blood collected on Day 1 (pre *IV* infusion) and Day 22 (post *IV* infusion). Heterophils and lymphocytes collectively made up >80% of the leukocytes enumerated. There was a numeric increase in percentage of monocytes from Day 1 to Day 22 for the control and treatment groups. When comparing pre- and post-infusion, monocytes increased in the 3 mL/kg group (*p*-value < 0.05). There were no other significant increases seen across the groups (Supplementary Table S1). Further, there were no noteworthy changes by infusion treatment in cell morphology (Supplementary Figure S3).

### 3.5 Enriched peripheral blood leukocyte cytospins: sub-acute trial

Enriched peripheral blood leukocytes were collected on Day 22. Heterophils and lymphocytes collectively made up >84% of the leukocytes enumerated. There was a numeric decreasing trend in percentage of monocytes with increasing *IV* honey that was not significant (*p* = 0.3783). Additionally, lymphocytes increased in the 3 mL/kg group compared to the control group (*p*-value < 0.05). Otherwise, there were no noteworthy changes in leukocyte morphology across groups (Supplementary Table S2 and Supplementary Figure S4).

### 3.6 Peripheral blood leukocyte cell surface markers: sub-acute trial

Three monoclonal antibodies were used to screen for changes in cell surface phenotypic expression of the leukocytes in the peripheral blood. Percentage of CD8+ T cell and Mono+ monocytic cells were not significantly different across treatment groups. There was a noteworthy numerical increase in IgM^+^ B cells in birds that received *IV* honey, which was insignificant (*p* = 0.4163; Table 3 and Supplementary Figure S5).

**Table 3:** Peripheral blood cell surface leukocyte phenotypic expression.

Cell surface marker differential	Control Median (min–max)	1 mL/kg Median (min–max)	3 mL/kg Median (min–max)
CD8	14.1 (9.1–18.3)	12.9 (8.5–29.3)	11.0 (9.1–12.1)
IgM	4.0 (2.5–5.1)	5.9 (2.9–11.9)	6.4 (3.8–7.8)
Mono	0.2 (0.1–0.3)	0.2 (0.1–0.6)	0.2 (0.2–0.3)

Peripheral blood cell surface markers from the sub-acute phase trial. *p* ≤ 0.05, Tukey’s test, Two-way ANOVA, *N* = ≥6 birds/treatment. Values highlighted in bold were significant from control values.

### 3.7 Peripheral blood plasma cytokine levels: sub-acute trial

Twelve chicken cytokines and chemokines were screened from plasma collected from birds on Day 22. Select cytokine/chemokine levels were relatively consistent, where others varied between pre and post *IV* infusions, but there was no significant trend based on honey infusion treatment. IL-16 significantly increased within the saline group, with a *p*-value < 0.05. However, IL-16 significantly decreased with the 1 mL/kg treatment group, with a *p*-value < 0.0001. IFNg significantly decreased in the saline and 1 mL/kg treatment groups, with *p*-values < 0.05. VEGF levels significantly declined in both the 1 mL/kg and 3 mL/kg treatment groups, with *p*-values < 0.05. No consistent treatment-related pro-inflammatory trends were identified (Table 4).

**Table 4:** Peripheral blood plasma cytokine levels: sub-acute trial.

Cytokine/Chemokine (pg/mL)	Control		1 mL/kg		3 mL/kg	
	Pre-infusion Median (min–max)	Post-infusion Median (min–max)	Pre-infusion Median (min–max)	Post-infusion Median (min–max)	Pre-infusion Median (min–max)	Post-infusion Median (min–max)
IL-16	159.6 (104.5–286.9)	**524.8**^**a**^ **(158.1–1,159)**	1,467 (1,084–1,954)	**508.6**^**b**^ **(388.4–744.4)**	346.8 (208.4–1,077)	439.5 (170.6–730.5)
IFN-α	5.5 (5.5–5.5)	5.5 (5.5–5.5)	5.5 (5.5–5.5)	5.5 (5.5–69.6)	5.5 (5.5–5.5)	5.5 (5.5–5.5)
IFNγ	16.6 (11.4–33.6)	**4.1**^**c**^ **(1.9–9.7)**	29.7 (14.9–43.6)	**4.8**^**d**^ **(1.9–29.7)**	19.4 (9.7–29.7)	6.4 (1.3–29.7)
IL-10	31.7 (31.7–31.7)	31.7 (31.7–31.7)	31.7 (31.7–197.6)	31.7 (31.7–144.0)	31.7 (31.7–60.6)	31.7 (31.7–31.7)
IL-21	34.3 (32.7–46.9)	29.5 (29.5–39.0)	38.2 (32.7–53.1)	32.7 (29.5–39)	38.2 (31.1–73.5)	32.7 (29.5–40.6)
IL-2	7.4 (7.4–112.3)	7.4 (7.4–23.2)	7.4 (7.4–7.4)	7.4 (7.4–7.4)	7.4 (7.4–7.4)	7.4 (7.4–7.4)
M-CSF	3,499.0 (3,030–4,308)	3,560.5 (2,895–3,722)	3,361.0 (2,564–4,237)	3,196.0 (2,581–3,826)	3,579.5 (3,280–4,035)	3,356.0 (3,019–3,787)
IL-6	0.3 (0.3–0.3)	0.3 (0.3–0.3)	0.3 (0.3–0.3)	0.3 (0.3–0.3)	0.3 (0.3–0.3)	0.3 (0.3–0.3)
MIP-3α	70.3 (50.8–85.9)	50.8 (41.8–180.3)	55.4 (41.8–161.6)	46.2 (29.5–91.3)	55.4 (50.8–70.2)	41.9 (29.5–193.0)
MIP-1β	62.7 (35.6–102.8)	97.1 (42.8–177.4)	87.7 (55.7–497.9)	161.8 (42.8–878.8)	99.0 (42.8–114.0)	100.9 (40.1–389.4)
RANTES	57.5 (32.6–130.7)	202.5 (27.8–357.1)	196.0 (100.1–409.2)	413.8 (107.0–995.0)	82.2 (54.1–680.4)	135.1 (27.8–326.5)
VEGF	10.8 (8.0–15.5)	7.1 (6.2–11.8)	11.8 (8.0–25.1)	**6.2**^**e**^ **(4.4–13.7)**	11.8 (6.2–21.2)	**7.1**^**f**^ **(4.4–8.0)**

Cytokine and chemokine levels measured from the sub-acute phase trial peripheral blood plasma samples. ^a–f^Values with different superscripts are significantly different, *p* ≤ 0.05, Bonferroni’s test, Two-way mixed effects model, *N* = ≥6 birds/treatment. Values highlighted in bold were significant from control values.

### 3.8 Histopathology: sub-acute trial

**Figure 2:**
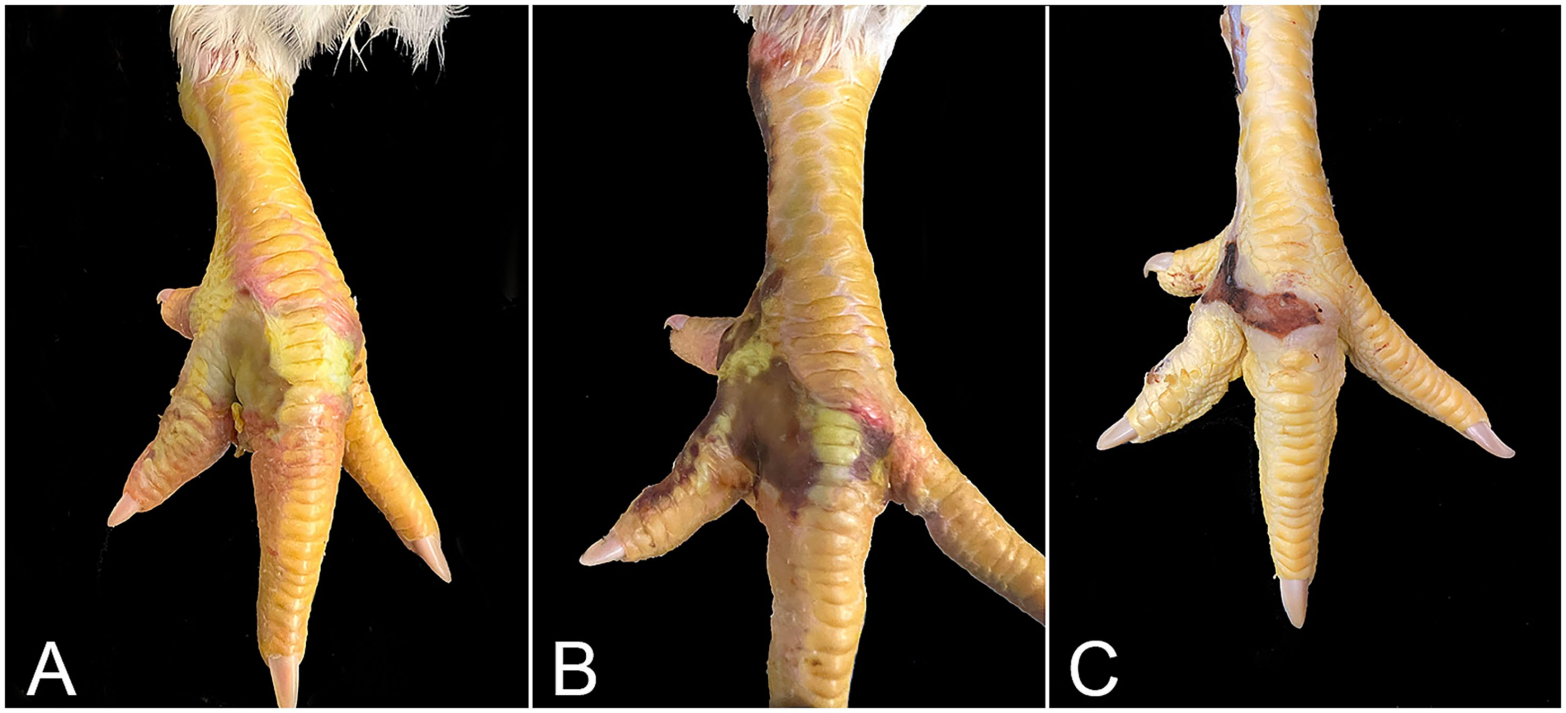
Images of phlebitis at administration site. Images of the bird in the 3 mL/kg *IV* filtered honey treatment with phlebitis on day 3 **(A).** Images of the leg during treatment with “honey bandages” on day 11 **(B)** and day 22 **(C)**.

Erosions overlying metatarsal veins occurred in 3/19 birds (16%). One 3 mL/kg bird developed severe localized phlebitis with fibrinoid necrosis (Figure 2), managed with topical honey bandages; this bird also showed elevated CK. No significant treatment-related lesions were seen in liver, lungs, or kidneys (Table 1). Incidental findings (lymphoid hyperplasia, granulopoiesis, pododermatitis) were common across groups and consistent with housing and prior study history. One bird in each trial had focal bronchopneumonia.

## 4 Discussion

This exploratory pilot study assessed tolerance of filtered raw honey administered *IV* to chickens. No overt acute systemic toxicity or consistent treatment-related immune alterations were observed. These findings align with mammalian and human IV honey studies showing good tolerability when administered slowly (10–13).

During the acute phase trial, no deaths were observed for any infusion dose group. Histopathologic lesions (fibrosis, pododermatitis, incidental hyperplasia) were observed but showed no dose-dependent pattern and were consistent with housing conditions and prior study use. Collectively, these findings suggest that chickens can tolerate acute *IV* honey infusions at doses up to 4 mL/kg of body weight.

Based on the results of the acute study, the sub-acute study proceeded with 3 treatment groups, control-saline, 1 mL/kg, and 3 mL/kg *IV* honey. One bird in the 3 mL/kg sub-acute group died after sedation on day 1 with ascites noted at necropsy. Given the chronic nature of ascites in broilers (23, 24) and timing prior to infusion, this event was not attributed to honey. Subsequent high-dose infusions were lengthened to 20 min as a precaution. Over the 22 days of the sub-acute phase trial, there was a positive weight gain across all treatment groups. Peripheral blood plasma chemistries compared between pre and post infusions showed an increase in total proteins and globulins across all treatment groups. This was likely attributed to the birds’ diet and positive growth conditions. It is worth noting that commercial analyzers are known to not be as reliable regarding these two avian values, possibly due to the calorimetric analysis that the rotors in the test use not being sensitive enough. For a more accurate assessment of total protein and globulins, the gold standard for measuring total protein and globulin in avians is electrophoresis. Immune parameters (cytology, phenotyping, cytokines) showed no pro-inflammatory trends attributable to honey. Clinical observations also did not show any significant negative side effects due to *IV* honey infusions.

Similar to the acute phase trial, gross examination and histologic evaluation of the chickens within the sub-acute phase trial revealed some abnormal findings, but did not appear to correlate with a specific treatment group. Of the 13 birds in the sub-acute study that received *IV* honey infusions, localized phlebitis was observed in one high-dose bird, but appeared related to possible extravasation rather than systemic effects (Figure 2). The phlebitis was successfully managed with “honey bandages,” which consisted of honey-soaked gauze wrapped with Vetrap bandaging tape, twice weekly until day of euthanasia. During the bandage changing process, the tissue site was evaluated to ensure proper healing. This bird did not display any other abnormal clinical signs for the duration of the study. This bird also had a noteworthy increase in CK, suggesting tissue damage at the site of extravasation.

However, several methodological limitations must be considered. The acute phase was severely underpowered (*n* = 2 per group), and sub-acute groups were small and uneven. Small sample sizes limit statistical power. Parametric testing assumptions may not have been fully met with ordinal/histologic data and low n; non-parametric approaches could be considered in future work. No formal power analysis or randomization was performed; birds were arbitrarily assigned. Sedation doses varied (2–4 mg/kg) based on subjective depth, and infusion durations differed (10 vs. 20 min) to mitigate fluid overload. Peripheral blood samples experienced a 12–16 h pre-analytical delay, and leukocyte viability was approximately 80%. These pre-analytical delays and variable sedation/infusions represent additional confounders. These factors, along with use of commercial broilers from a prior nutrition study (rather than specific-pathogen-free birds), may have influenced results. Larger, randomized studies with pathogen-free birds and standardized protocols are needed.

In summary, this preliminary study did show that *IV* honey infusions can be administered to birds without causing widespread mortality or severe immediate systemic effects. This is viewed to be favorable as it is not desired to provide an adjunct therapeutic to treat a proinflammatory disease when it alone has the potential to function as a proinflammatory agent. Based on the literature in other species, *IV* honey does appear to have positive medicinal applications. Still, additional studies are needed to assess dose to frequency and duration of treatment as well as direct efficacy as an adjunct treatment in birds with morbidities (i.e., cancer, infections).

## 5 Conclusion

Under the conditions of this pilot study, broiler chickens tolerated a single *IV* infusion of filtered raw honey up to 4 mL/kg and three sub-acute infusions up to 3 mL/kg without overt adverse systemic effects or consistent immune activation. These data suggest *IV* honey may be a feasible therapeutic option in birds, but further controlled studies are required to confirm safety, optimize dosing, and evaluate efficacy in diseased animals.

## Data Availability

The original contributions presented in the study are included in the article/Supplementary material, further inquiries can be directed to the corresponding author.
