# Supplementary material for: Tolerance of chickens to acute and sub-acute intravenous administration of raw filtered honey
Source: Front Vet Sci. 2026 Jul 14;13:1832182. doi: 10.3389/fvets.2026.1832182 (PMC13407112; doi:10.3389/fvets.2026.1832182)

Supplementary Figure S1: Acute Phase Trial Histopathology

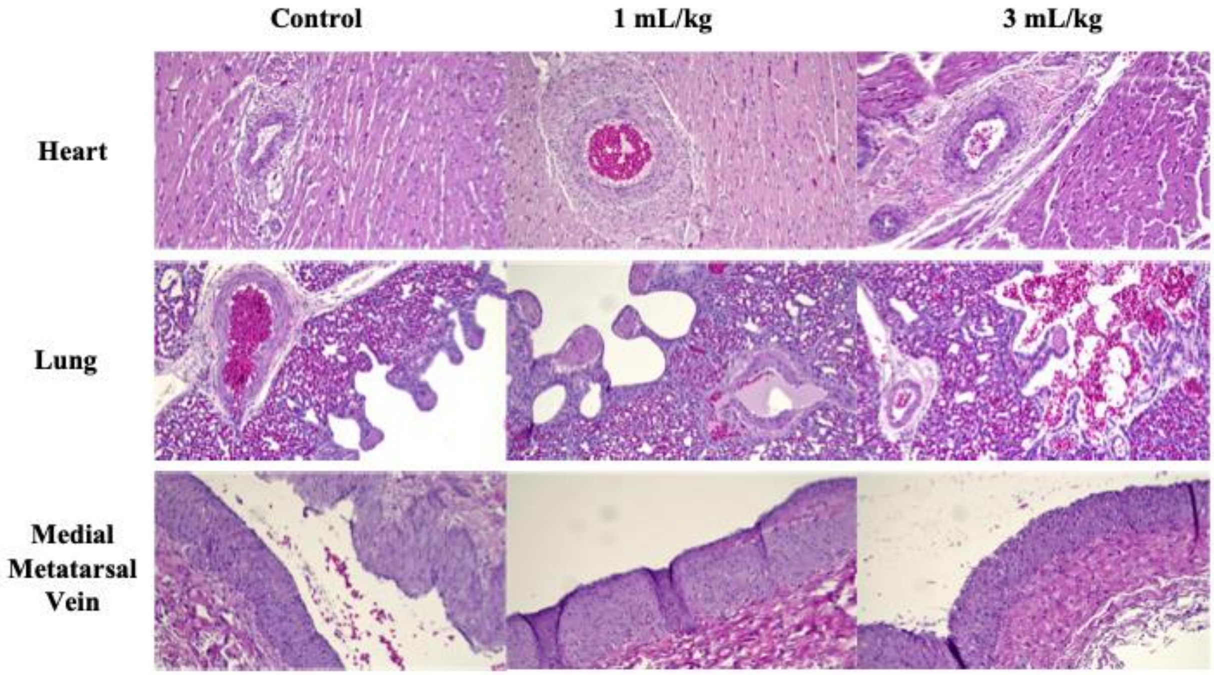

Legend. Representative images from tissue samples for the treatment groups. There were no remarkable findings associated with a treatment group found on histopathology.

Supplementary Figure S2: Bird Weight Gain: Sub-acute Phase Trial

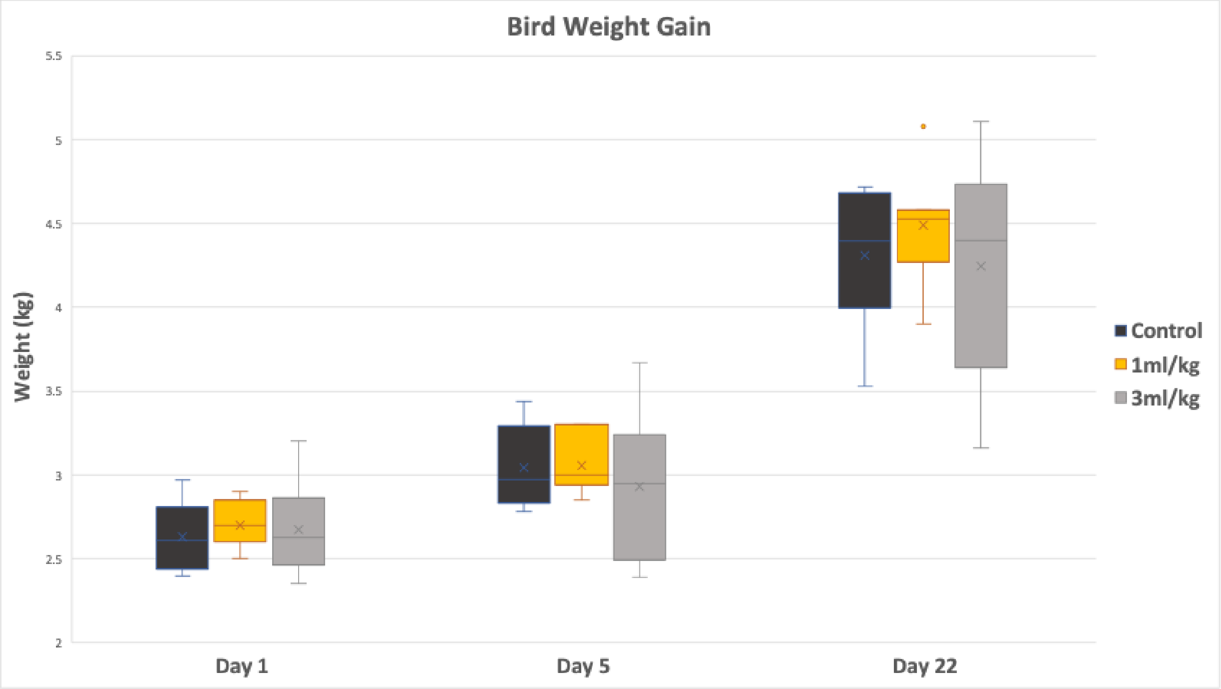

Legend. Weights taken throughout the sub-acute phase trial reveal a positive weight gain across all treatment groups.

Supplementary Table S1: Peripheral Blood Smears Cytology: Sub-acute Phase Trial

	**Control**		**1 mL/kg**		**3 mL/kg**	
**Leukocyte 5-point differential (%)**	**Pre-infusion** Median (Min/Max)	**Post-infusion** Median (Min/Max)	**Pre-infusion** Median (Min/Max)	**Post-infusion** (Median (Min/Max)	**Pre-infusion** Median (Min/Max)	**Post-infusion** Median (Min/Max)
**Heterophils**	39 (31-54)	44.5 (29-50)	40 (25-53)	40.5 (28-56)	47 (21-55)	29 (26-55)
**Lymphocytes**	47.5 (27-60)	43.5 (21-51)	45.5 (37-60)	44.5 (34-57)	42 (38-72)	47 (24-60)
**Monocytes**	10 (2-18)	12 (6-29)	9.5 (1-21)	10.5 (5-24)	5 (4-7)	**15** [a] **(10-24)**
**Basophils**	0.5 (0-13)	2.5 (0-5)	3 (2-6)	1 (0-2)	3 (0-6)	4 (1-6)
**Eosinophils**	0 (0-5)	0 (0-0)	0 (0-3)	0 (0-0)	0 (0-0)	0 (0-0)

[a] Value with superscript is significantly different, $p \leq 0.05$, Bonferroni's test, Two-way mixed effects model, N= ≥6 birds/ treatment

Supplementary Figure S3: Peripheral Blood Smears Cytology: Sub-acute Phase Trial

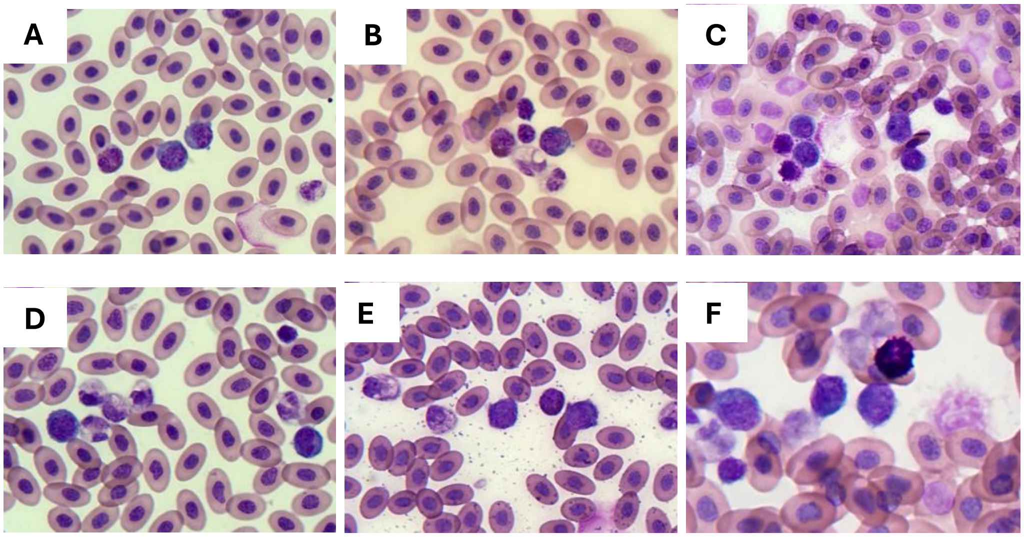

Legend. Sub-acute phase trial whole blood 5-point differentials representative images. A) Day 1: Control group B) Day 1: 1 mL/kg treatment group C) Day 1: 3 mL/kg treatment group D) Day 22: Control group E) Day 22: 1 mL/kg F) Day 22: 3 mL/kg treatment group. No consistent changes in cell morphology or evidence of reactive lymphocytes were noted in any of the treatment groups.

Supplementary Table S2: Enriched Peripheral Blood Leukocyte Cytospins: Sub-acute Phase Trial

Leukocyte 5-point differential (%)	Control<br><br>Median (Min-Max)	1 mL/kg<br><br>Median (Min-Max)	3 mL/kg<br><br>Median (Min-Max)
**Heterophils**	43 (38-49)	40 (33-42)	35 (32-48)
**Lymphocytes**	42.5 (27-52)	50 (38-53)	54 (40-58)
**Monocytes**	9.5 (4-27)	10 (4-16)	8 (6-11)
**Basophils**	3.5 (1-5)	4 (1-6)	4 (1-7)
**Eosinophils**	0 (0-0)	0 (0-0)	0 (0-0)

$p \leq 0.05$, Tukey's test, Two-way ANOVA, N= ≥6 birds/ treatment

Supplementary Figure S4: Enriched Peripheral Blood Leukocyte Cytospins: Sub-acute Phase Trial

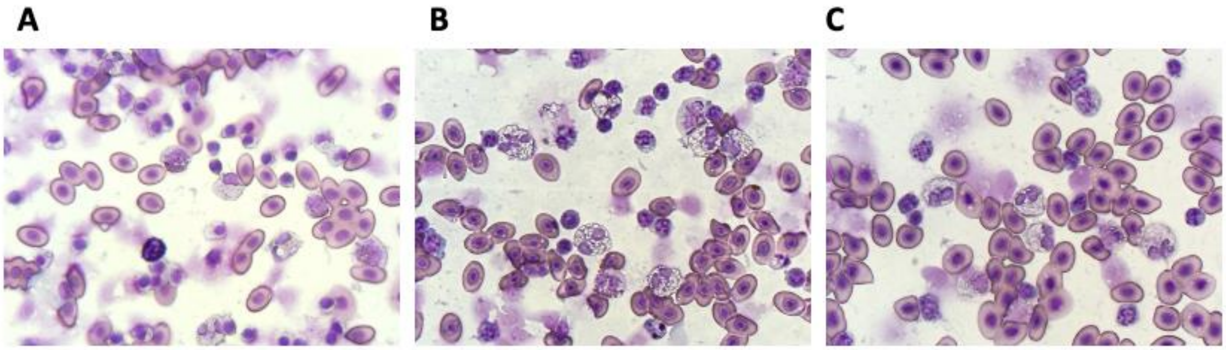

Legend. Sub-acute phase trial enriched peripheral blood leukocyte cytospins differentials representative images. A) Control group B) 1 mL/kg treatment group C) 3 mL/kg treatment group. No statistically significant differences were observed between treatment groups.

Supplementary Figure S5: Peripheral Blood Cell Surface Leukocyte Phenotypic Expression Gating

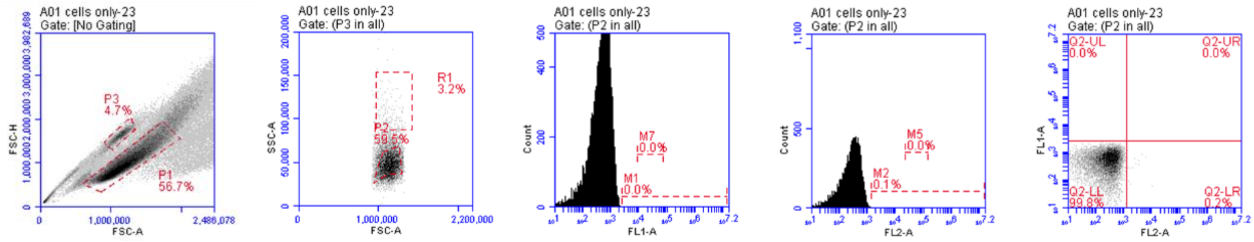

Supplement: Supplementary file 1 [file Data_Sheet_1.PDF]
